# Dr. Samuel W. Neall

**Published:** 1884-05

**Authors:** 


### OBITUARY.

### DR. SAMUEL W. NEALL.

Died at Camden, N. J., on March the 29th, 1884. DR. SAMUEL W. NEAL, aged 76 years.

Dr. Neall belonged to that class of old-time dentists that is rapidly passing away, and he deserves more than ordinary notice. He was born at Milford, Delaware, February 10th, 1808, and came to Philadelphia at the age of sixteen. In his early life he followed the occupation of a printer until thirty years of age, when he studied dentistry with the firm of Harbert & Neal in this city. He graduated under them, and, according to the custom in vogue in ante-dental college days, received their diploma, which was kindly shown us by his son, a quaint piece of parchment, printed with gold foil, (done by Dr. Neall himself), stating that "he had concluded his studies, &c.. was well prepared in the branches of practical and operative dentistry ; the *modus operandi* of setting teeth according to modern style ; and the *manufacture of porcelain and mineral teeth.*" This was dated February 18th, 1838, and signed Harbert & Neall, 303 Arch Street. In 1840 he first commenced the manufacture of artificial teeth, and was the second oldest manufacturer for the general market in the country ; Samuel W. Stockton being the first. He followed the dual occupation of dentistry and manufacturer in this city until 1862, when he relinquished the former but continued manufacturing—part of the time in conjunction with his son—until March, 1881, when he gave it up entirely, and his son became his successor. He enjoyed his retirement but a little over two years, when he was taken with the sickness—heart disease and dropsy. That terminated in his death.

Dr. Neall was the first manufacturer of teeth that "put the gum enamel in the mould," the others adhering to the old practice of using a brush for some years. But one by one they gradually adopted his system, which is now universally used. Dr. Neall was a most excellent operator, a fine mechanic, and a careful, quiet laborious man. By a blameless, industrious life he acquired a competency, made many friends, and the science of dentistry is none the worse that he has left upon her the impressions of his handiwork.—*Dental Office and Laboratory.*

